# Shifting the Paradigm: Antiracist Education for Advanced Practice Nursing Providers

**DOI:** 10.1089/heq.2023.0088

**Published:** 2023-09-13

**Authors:** Lisa Mihaly, Teresa Scherzer, Cara McGuinness, Linda Stephan

**Affiliations:** ^1^ Department of Family Health Care Nursing, School of Nursing, University of California, San Francisco, San Francisco, California, USA.; ^2^ Division of Adolescent and Young Adult Medicine, Nursing Faculty, School of Medicine, University of California, San Francisco, San Francisco, California, USA.; ^3^ Office of the Dean, School of Nursing, University of California, San Francisco, San Francisco, California, USA.; ^4^ Department of Obstetrics and Gynecology, Boston University School of Medicine/Boston Medical Center, Boston, Massachusetts, USA.

**Keywords:** education, structural, racism, advanced practice nursing, antiracism

## Abstract

**Introduction:**

Racism in the United States adversely impacts health outcomes. Achieving health equity will require an explicitly antiracist approach to the education of health care providers (HCPs). This article examines a required course that focuses on teaching advanced practice nursing students about the structural foundations of racism. This approach shifts significantly away from teaching race-based medicine (which assumes a biological basis for disparities) and the social determinants of health (which often blames individuals for disparities).

**Methods:**

A mixed methods evaluation was conducted to understand the change in learners' understanding of (1) structural racism and (2) the role that HCPs can play in addressing structural racism. Anonymous surveys asked the following: (1) What are three examples of structural racism in the context of health care? and (2) What is the HCP's role in addressing structural racism?

**Results:**

Statistically significant increases were observed. The percentage of students who could provide at least one example of structural racism increased from 41% to 70%. Significant increases were also found in students' abilities to identify structural and institutional antiracist interventions.

**Discussion:**

This project yields important data that can inform educational efforts focused on structural racism. The results strongly suggest that the course resulted in a change in student understanding of racism in health care and strategies to address it.

**Health Equity Implications:**

The development of a required course for advanced practice nurses focused on structural racism, including attention to social and institutional interventions, can significantly shift HCP understanding and is one strategy to move us toward health equity.

## Introduction

Health equity has been an articulated goal of health care providers (HCPs), health care systems, and governments since the mid-20th century.^1^ The World Health Organization defines health equity as the “absence of unfair, avoidable or remediable differences in health among population groups, defined by social, economic, demographic or geographic characteristics.”^2^ In the United States, we are very far from an equitable distribution of resources, and health outcomes can be both predicted by zip code and can be differentiated by race.^3,4^ The health of racialized and minoritized people in the United States is significantly worse than the health of white people on many metrics. An exhaustive list is beyond the scope of this article, but a few illustrative examples include the following:

- Black women in the United States are two to three times more likely to experience serious morbidity or to die in childbirth than their white counterparts;^5^
- Black children in the United States are twice as likely to experience severe asthma than their white counterparts;^6^
- Black, Indigenous, and People of Color individuals in the United States are two times more likely to die from complications of COVID-19 than their white counterparts.^7^

In the education of HCPs, there have been many attempts to explain these differences in health outcomes and to provide health care trainees with strategies to disrupt these inequities. The concept of social determinants of health (SDOH) as an explanation for health inequities dates back to at least 1967 with the Whitehall Study.^8,9^ While the study of SDOH—the conditions of daily living that shape the health of an individual and of the population—is integral to medical and nursing education, it is incomplete.^2,10^

Examining only the SDOH stops short of addressing the larger upstream political and social forces that determine the conditions in which people are born, live, work, and grow—the Structural Determinants of Health.^10–12^ Further, when the SDOH model is applied in health education and health care in isolation, HCPs risk blaming the individual and focusing too much energy on changing individual-level behaviors versus changing the systems and structures that are the root cause of the inequities observed.^12,13^

Attempts to improve the interactions between patients and providers to support greater health equity began in the late 1980s, as health education pedagogy focused on the concept of cultural competence. The cultural competency model teaches providers that they can deliver better care if they study their patients' cultures.^14^ In the late 1990s, “cultural humility” emerges in the literature, with an increased focus on provider self-evaluation and critique.^15^ The models of cultural competency and cultural humility train HCPs to develop “linguistic competencies” and focus on culturally sensitive, nonjudgmental ways to build rapport with patients. In the 20 years since the development of concepts of cultural competency and cultural humility, training in these areas has helped promote consideration of the impact of stigma and bias on treatment decisions at the individual patient level.

However, focusing on provider–patient communication and individual behaviors misses the structural elements at play, such as racism and regressive policies that keep certain populations in poverty, results in inequitable health care access, and leads to discriminatory zoning policies. Public health, social science, and critical race studies scholars have, for many decades, located discrimination not just in the attitudes of individual persons but also in the actions of institutions, markets, and health care systems.^13,16,17^ In 2014, “structural competency” emerged in medical education literature.^18^

Structural competency refers to a HCP's trained ability to discern how a host of issues defined clinically as symptoms, attitudes, or diseases (e.g., depression, obesity, hypertension, smoking, medication “noncompliance,” trauma, psychosis) is the result of a number of upstream decisions regarding health care and food delivery systems, zoning laws, urban and rural infrastructures, and even about the very definitions of illness and health.^11,18,19^ Structural racism is defined as the policies, economic systems, power dynamics, and social hierarchies that have produced and maintained inequities along the lines of race in the United States.^19,20^ The concept of structural racism represents a paradigm shift for many students—a shift away from what they have been taught in their previous education and what they have absorbed from cultural norms.

Several interlocking factors combined to drive the development of a course focusing on structural racism. These factors include the following: (1) The cultural and pedagogical shift in medical education from a focus on individual factors to an understanding of structural forces as a driver of health inequities, (2) pressure from students to develop a more progressive curriculum, and (3) changes in national guidelines for training of advanced practice nurses.^21^ The course we developed for our school's Master's nursing program—Racism, Healthcare, and Social Justice—piloted in 2021 and in 2022, became required for all advanced practice nurses educated at our institution.

The course uses a structural competency framework and focuses on structural racism, specifically addressing the policies, historical precedence, economic systems, power dynamics, and social hierarchies that have produced and maintained inequities along the lines of race for individuals living in the United States. This course also explores ways that HCPs can address structural racism, with a focus on antiracist interventions targeted at the institutional and societal levels.

We conducted a mixed methods evaluation of learners enrolled in this course to understand the change in learners' understanding of (1) structural racism and (2) the role HCPs can play in addressing structural racism.

## Methods

The setting for this study is a school of nursing affiliated with a large academic medical center in California. The demographics of all Master's students during the academic year 2021–2022 were 7% Black, 27% Asian, 15% Latinx, 37% White, and 86% Female. The course met for 10 weeks synchronously through Zoom for 2 h. During the synchronous class time, faculty and expert guest speakers provided a 60- to 90-min lecture.

Examples of content covered in the course include social justice, race-based medicine, critical race theory, reproductive justice, xenophobia, environmental racism, and the decolonization of medicine. Following each lecture, the course faculty facilitated large group discussions for 30–60 min. Students were expected to review required readings and other media content before attending class related to the weekly course content. They also submitted an anonymous response to a specific reflection question before attending class each week, and a second response to a reflection question after each class session. Faculty synthesized themes from student reflections and shared them with students weekly.

To evaluate the learning from this course, we created two online surveys to use in a pre-test and post-test design, focusing on two open-ended items common to both surveys. To ensure a high response rate, we asked students to complete the surveys during class time. To encourage honest responses and for purposes of confidentiality, the survey was anonymous. Our project is covered by one author's (Scherzer) Institutional Review Board protocol for educational evaluation. The first survey was conducted during the first class meeting, before the delivery of course content; the second survey was conducted at the end of the last day of class. This article analyzes two open-ended questions asked in both of those surveys: (1) What are three examples of structural racism in the context of health care? and (2) What is the HCP's role in addressing structural racism?

Qualitative data for the above two questions were coded using coding schemes that allowed us to categorize the different types of responses and create dichotomous variables. We then were able to conduct chi-square tests to compare pre and post-test results and to assess whether differences were statistically significant.

### Student ability to provide examples of structural racism in the context of health care

The first question asked students to provide examples of structural racism. To evaluate participant responses, we constructed codes analogous to quantitative answer choices that indicated one or more of the following: one, two, or three strong examples of structural racism. For text to be coded as an example of structural racism, it had to contain an explicit description of policy or practice that fosters or reinforces racial inequity or white supremacy (e.g., redlining or other social policies that lead to inequitable distribution of resources, scientific racism that informs academic systems, educational materials or medical practice that inappropriately use race as a biological construct). Answers that were not examples of structural racism were coded based on categories that emerged from the data: (1) SDOH or other downstream effects of structural racism, (2) personally mediated racism, (3) unclear, and (4) do not know (see Table 1 for examples).

**Table 1.** Coding Student Responses to “What Are Three Examples of Structural Racism?”

Code	Definition used for coding	Examples of student responses (verbatim)
Personally mediated racism	“Private prejudices held by individuals” expressed through attitudes and behavior^17^	The way in which HCPs view patients when walking into the room. Based on the color of their skin, they already have an idea in their mind about what is wrong with the patient. Providers refusing to acknowledge that racism still exist and is a social determinant of health. This invalidates the struggles and oppressions of black and indigenous peoples, which is inherently tied to their overall health and wellbeing. Implicit bias, implicit prejudice, and stereotyping leading to unequal treatment. Clinicians not bothering to explain things to patients believed unable to understand/care. Assumptions of a patient's health literacy based on socioeconomic background [or] not listening to a patient's complaint or being dismissive not treating pain appropriately (i.e., under treating pain for POC). Assumption of “noncompliance” without regard to access to health care
SDOH and other downstream effects of structural racism	“The social determinants of health (SDH) are the nonmedical factors that influence health outcomes. They are the conditions in which people are born and grow, work, live, and age, and the wider set of forces and systems shaping the conditions of daily life.” SDH includes, but is not limited to education, food access, employment status, income levels, working conditions, housing, access to health care^24^	Hospitals located only in [high] resourced areas; clinics being open only during hourly worker hours (male–female 9–5). Employment that exposes workers to environmental toxins and unsafe working conditions leading to negative health outcomes. Food deserts—lack of access to healthy foods in minority neighborhoods. Language barriers in health care—lack of non-English speaking providers. Underrepresentation of BIPOC folks in health care perpetuates the historical representation of what a health care professional is limits upward mobility of those persons of color that do make it into the profession. Unequal access and cost to health care and mental health care (insurance being tied to jobs or insurance costs tied to comorbid conditions). African American women more likely to die during childbirth. Lack of medical research on diverse populations. Access to quality health care services-access to a safe home/neighborhood/physical environment-access to healthy food and clean water
Structural racism	Policies, economic systems, power dynamics, and social hierarchies that have produced and maintained inequities along the lines of race^19,20^	Textbooks assigned to health/medicine programs that purport unfound racial biases. Three examples of structural racism in the context of health care include housing policy, environmental policy, and educational policy. These types of policy impact delivery of health care, the types of health insurance that patients have, and the type of illness that patients are afflicted by. Within medicine, race-based medicine is an example of structural racist forces having impact on health care policy and delivery. Policies rooted in eugenic movement like mass sterilizations, systemic disinvestment in minority communities leading to underfunded facilities with fewer clinicians. Medical providers are taught that race is based in biology (e.g., race-based parameters for eGFR or lung capacity); Hospitals use structured decision-making tools for drug testing pregnant people that disproportionately result in drug testing/child separation for Black and Indigenous birthing people. The United States chronically underfunds and understaffs the Indian Health Services. Political disenfranchisement and disempowerment through voter suppression laws and gerrymandering; environmental injustice where people with less means must live in areas of higher pollution and less access to clean outdoor spaces; and criminal injustice where people of color are disproportionately detained and given more stringent consequences for lesser crimes than their white counterparts. Race-based algorithms. Thinking of race as biological rather than a social construct. Structural racism exists through redlining and the creation of food deserts and lack of investment in communities of color, which lead to poor health outcomes. Structural racism exists in policy that does not support distribution of resources. Policies formulated by those in positions of privilege, hiring practices that disadvantage people of color, passive behavior by leadership when people speak up
Unclear		Statistics based on race. Trust. Latinx expect families to help with recovery however, many hospitals delegate recovery assistance to strangers, i.e., nurses and HCP. Medications in general and I refrigerated meds like insulin are really difficult for the unhoused to manage. The terms “compliant and uncompliant” infer that patients want to or I want to be healthy. Accessibility, ability to pay (insurance), patient outcomes. Any use of the word “pre-contemplative.” The medical hierarchy of doctors over nurses over aids, etc. Westernized medicine in itself. Insurance differences in what they cover. Power dynamics. Economic based care. Policies, legislature. How medication is accessed, who can access it and how payment is structured. Socioeconomic, environmental, housing, environment, and immigration status
Do not know		Not quite sure. I know it can lead to discrimination against patients, but I just can't imagine treating a patient differently based on the color of their skin. Good thing I am taking this class! Don't know

BIPOC, Black, Indigenous, and People of Color; eGFR, estimated Glomerular Filtration Rate; HCP, health care provider; POC, People of Color; SDOH, social determinants of health.

Each respondent was assigned a code for every answer that they submitted. We asked for three examples—some students provided more and some provided fewer. If a respondent provided only three examples of structural racism, they were assigned a single code. Alternatively, if a respondent submitted one example of structural racism, one example of SDOH, and one answer that was unclear, we assigned that respondent three codes (structural racism, SDOH, and unclear). As we show in the Results section, a substantial percentage of respondents provided answers that received multiple codes, including “unclear.”

Table 1 illustrates our codes and definitions, and a selection of answers that students provided as examples of structural racism from pre-test and post-test data.

### Student ability to identify the HCP's role in addressing structural racism

The second question asked respondents to describe the role of the HCP in addressing structural racism. We developed a “levels of intervention” coding schematic based on the “levels of antiracism” in Harper Browne and O'Connor's “social-ecological model of racism and antiracism.”^22^ We coded responses from pre-test and post-test using the model's levels of antiracist interventions and definitions: Systemic/Societal, Institutional/Community, Interpersonal, and Intrapersonal (Table 2). As we did for the first question, we applied multiple codes to responses that addressed multiple levels, and we added a code for an unclear response or response that was not explicitly related to structural racism. We also applied two additional codes to describe themes that emerged from the data: “Colorblind” and “Cultural Competence/Humility” (Table 2).

**Table 2.** Levels of Intervention: Coding Student Responses to “What Is the Role of the Health Care Provider in Addressing Structural Racism?”

Code	Definition used for coding^22^^,^^a^	Examples of student responses (verbatim)
Intrapersonal/individual	Personal reflection to counteract one's own racialized ideas, feelings, and attitudes, including: • Understanding how one's own racial socialization and identity have shaped and been influenced by personal life experiences • Examining the personal impact of living in a racist society	Understanding on bias, stereotypes, and remaining mindful in how that impacts perception of patient and decision making. Checking one's own biases. Continue to be conscious and aware of the impact of structural racism and its deep history, continue to educate myself, colleagues, and community, listen to patients' stories and work with them from their expectation and perspective, while continuing with evidence-based practice.
Interpersonal/relational	Verbal and nonverbal interactions between individuals, characterized by: • Appreciation for each person's unique identity, experiences, and perspectives • Not making or acting on assumptions about a person based on perceived race, ethnicity, national origin, gender, sexuality, or disability • Willingness to apologize and/or make reparations for harm caused	Provide care that is patient centered and individualized. To make sure that their care and history is a full assessment because each patient has hurdles and struggles. This class has opened my eyes to how important it is to be mindful of what our patients come with and what is preventing them to achieve their health goals. From an individual standpoint, it is taking that time with each patient to determine what their needs are and how you can best work with them and their life for the best outcomes, as well as creating a safe and welcoming space for all people.
Institutional/community	Recognizing and eliminating discriminatory policies, procedures, and practices in organizational and community contexts to create and sustain equitable access to power, privilege, opportunity, and resources, characterized by • Settings that are accessible, welcoming, and affirming to all • Assessment of policies, procedures, and practices that lead to disparate outcomes • Adoption of new policies, procedures, and practices to replace or counteract harmful ones and to repair harm done • Swift responses to racist actions or display of bias	Advocating for and speaking out when they experience practices being done in the institution they work in that may seem to be discriminating about people or specific populations based on their ethnicity, race, language, etc. To create safe spaces for people of color in institutions and to help them build more power in themselves and their communities.
Systemic/societal	Ideology, values, norms, laws, policies, and practices that create and sustain equitable access to power, privilege, opportunity, and resources within and across the functioning of systems and in their outcomes, characterized by: • Valuing and protecting the fundamental humanity and rights of all people • Acknowledgment of the racist roots of laws, policies, and systems that result in disparate outcomes—and specific, targeted efforts to redress and counteract them • Commitment to directing resources and supports to those who have been harmed by racism • Developing and implementing new strategies to achieve goals like public safety and child protection • Leadership by and alongside individuals and communities who have been harmed	It is our role to go beyond just treating our patients individually, but to go after dismantling systems of colonization, getting involved in policy, engaging with our communities, working in activism. Advocate for system-level and policy-level changes to mitigate institutional racism. Advocating and centering communities most impacted by structural racism. To speak up about structural racism when it is seen in systems, to advocate for policies that dismantle racist systems. Know your history, think systemically and upstream—why do these racists policies exist? Advocacy and policy change! Unionize! Organize! We need to be actively involved in speaking against practices that are rooted in racism, the third is being involved on a legislative and political level to bring perspective to how policies and procedures can be made or changed. A provider should also join a national nursing organization and advocate for social justice and influence lawmakers.
Other codes		

**Code**	**Definition**	**Examples of student responses (verbatim)**
Cultural competence/cultural humility		Taking the time to learn about patients' backgrounds before implementing treatment plans. Being culturally aware and sensitive. The HCP must remain up to date with common cultural practices seen in their specific clinical setting.
Colorblind	“Colorblind” Ideology focuses on individuals and posits that everyone should be treated equally no matter what their “race.” It does not recognize racism as a system that reinforces white supremacy through practices such as police brutality and redlining^25^	To provide equitable care to all. provide judgment-free care. Identifying personal biases and treating a patient as an individual, not based on race. The role of the HCP is to see the patient regardless of race. To treat them based on your examination and history of them and not based on preconceived notions or stereotypes that society places on us. The HCP's role in addressing structural racism is to use individual actions to inform systems of change, to educate their peers, to educate themselves, and to constantly evaluate themselves to not harm patients
Unclear		The HCP's role is to not assign blame to patients for their health conditions, and to counteract structural racism by providing access to resources that can combat some of the systemic factors that an individual may be experiencing. HCPs have ethical responsibilities to serve patients and with that play a vital part in seeing that structural racism is mitigated in the community. To be aware and actively work to bring attention and recognition and work to improve treatment and outcomes to make them equal. Destabilize structural racism when encountered

^a^ Used with permission by the authors (e-mail correspondence November 29, 2022).

Data analysis used Stata 17.^23^ Since our variables were dichotomous, we conducted chi-square tests to compare the proportion of respondents in pre-test and post-test for each constructed answer choice and to assess whether those differences were statistically significant.

## Results

Of the 189 students enrolled in the course, we received 167 responses for the pre-test and 170 for the post-test. We excluded submitted surveys that were <67% complete, resulting in 157 pre-test and 162 post-test surveys (83% and 86% response rates).

### Examples of structural racism

As shown in Table 3, chi square tests demonstrated significant increases between the proportion of respondents in pre-test versus post-test, who provided three or two examples of structural racism. The percentage of respondents providing three examples of structural racism increased approximately fivefold, from 3% to 16%. The percentage of respondents providing two examples tripled from 9% to 27%. Overall, the percentage of respondents who were able to provide at least one example of structural racism increased significantly from 41% to 70%.

**Table 3.** What Are Three Examples of Structural Racism?

	Pre-test (157), ***n*** (%)	Post-test (162), ***n*** (%)	Pearson's ***χ***^2^	***p***
Provided 3 examples	4 (3)^a^	26 (16)	17.06	0.0000
Provided 2 examples	14 (9)	43 (27)	16.88	0.0000
Provided 1 example	47 (30)	45 (28)	0.18	0.6710
Provided 1 or more examples	65 (41)	114 (70)	27.17	0.0000
Other responses				
Personally mediated example	45 (29)	42 (26)	0.30	0.5830
SDOH example	104 (66)	103 (64)	0.24	0.6190
Unclear response	78 (50)	51 (31)	10.97	0.0010
Don't know	5 (3)	0	5.24	0.0220

^a^ The percentages in each column will not total 100%. Except for respondents who provided three examples of structural racism, each respondent was assigned a code for each answer they submitted.

In both surveys, a substantial percentage of respondents cited an example of personally mediated racism (29% pre-test and 26% post-test) and/or SDOH (66% and 64%) as at least one of their examples of structural racism. While both surveys had a substantial percentage of respondents who submitted at least one answer that was unclear or not explicitly tied to structural racism (50% and 31%), this type of response decreased significantly in the second survey. No respondent submitted a “do not know” answer in the second survey.

### The role of the HCP in addressing structural racism

Table 4 shows the significant differences between pre-test and post-test in respondents' perceptions of the role of HCPs in addressing structural racism. The proportion of respondents who listed societal (9–26%), institutional (24–46%), and interpersonal (17–27%) antiracist interventions rose from pre-test to post-test. The proportion of respondents who listed an intrapersonal (46–27%) antiracist intervention decreased from pre-test to post-test. All these differences were statistically significant.

**Table 4.** What Is the Health Care Provider's Role in Addressing Structural Racism?

	Pre-test (157), ***n*** (%)	Post-test (162), ***n*** (%)	Pearson's ***χ***^2^	***p***
Systemic/societal	14 (9)	42 (26)	15.94	0.0001
Institutional/community	37 (24)	75 (46)	18.08	0.0000
Interpersonal	27 (17)	43 (27)	4.07	0.0438
Intrapersonal	72 (46)	44 (27)	12.05	0.0005
Other responses				
Cultural competence/cultural humility	8 (5)	2 (1)	3.91	0.048
Colorblind	22 (14)	12 (7)	3.65	0.0560
Unclear	53 (34)	48 (30)	0.63	0.428

The proportion of respondents answering with “colorblind” or “cultural competence”-related roles decreased in the second survey. Both surveys had ∼30% of respondents who submitted at least one answer that was vague or unclear about the role of the HCP in addressing structural racism.

A number of respondents' answers touched on multiple levels of intervention (data not shown). The proportion of respondents whose answers referred to both systemic/societal and institutional/community interventions increased significantly from pre-test to post-test, from 4% to 20%, respectively (*p*=0.0000).

## Discussion

HCPs need to understand structural racism to provide high-quality care and move toward health equity. This project yields intriguing and important data that can inform nursing and medical education's efforts to focus on structural racism, as the results strongly suggest that this course resulted in a shift in students' understanding of structural racism in health care and antiracist strategies that HCPs can employ to address structural racism.

The ability to provide an example of structural racism reflects students' abilities to apply the concept to their lived and educational experiences and demonstrates some understanding of the concept of structural racism. This study finds statistically significant shifts in the number of students who could provide examples of structural racism—specifically, this study found a fivefold increase in the number of individuals who could provide three examples of structural racism and a threefold increase in the number of individuals who could provide two examples by the end of the course.

In addition, an ability to describe antiracist interventions at the societal and institutional levels reflects students' abilities to understand that structural racism needs to be addressed by changing the upstream societal and institutional level structures that create inequities. This study found a significant increase in the number of students who were able to name antiracist interventions at the societal and institutional levels by the end of this course. These findings suggest that this course can effectively change learners' understanding of the concept of structural racism in health care and how learners think about targeting their antiracist interventions.

This study also found, at the end of the course, that 29% of students were still unable to articulate at least one example of structural racism. Some potential factors may contribute to this finding. Students clearly could not be graded on their survey responses, and some wrote fragments or very unclear sentences, which limited our ability to interpret some of the data. However, more likely, this finding may be a result of students being exposed to concepts that directly conflict with the content in this course. For example, while this class explicitly taught that race-based medicine is not medically sound (because race is a social construct, not a biologically meaningful one), other concurrent courses in the Master's Program continue to teach race-based medicine.

Changing the paradigm in which students address racism in health care requires that the concepts of structural racism are embedded in the entire curriculum, not just a single course, and that racist concepts integral to clinical care are dismantled in all courses in the curriculum, not a single course. As we expected, this finding suggests that a single course, while being a great step forward, is insufficient to change the paradigm and dismantle structural racism in the educational setting.

### Limitations

There are several limitations of this study. First, students completed surveys anonymously—therefore, we cannot link student responses to demographic data, and so we are unable to discern if there are racial, ethnic, or gender differences in student understanding of the content or their change in understanding over time. The anonymity also means we cannot compare any specific learner's pre-test and post-test answers.

We also do not know if this course will change students' actions as learners and future HCPs, especially given the hierarchical context of health care delivery and training, and the fact that textbooks and literature used in the broader curriculum utilize race as a biological construct, which is contrary to concepts taught in this course. This is a potential research question that may be able to be addressed by surveying or interviewing these students in the future. It is also hard to quantify the actual impact of this course on population health and health disparities. We hope to explore this in the future by asking these learners about antiracist activities that they have engaged in and the impact those activities have had on patients, colleagues, and organizations.

## Health Equity Implications

*Racism, Health Care, and Social Justice* is a single course that is one piece of an antiracist approach to the education of HCPs. We are well aware that advancing health equity requires a broad and deep approach. We found that the course is a single intervention that moved the needle of student understanding of structural racism and their intent to incorporate antiracist actions into their advanced practice nursing roles. A curriculum that embeds antiracist concepts into all courses would almost certainly help students develop a more nuanced understanding of these very complex topics. In the very hierarchical context of health care, students and new providers cannot, alone, educate (and re-educate) their superiors. Further, textbooks and literature that address race as a biological construct—a linchpin of structural racism—should be reviewed and updated.

## Abbreviations Used

BIPOC: Black, Indigenous, and People of Color
HCP: health care provider
SDOH: social determinants of health
